# A few-emitter solid-state multi-exciton laser

**DOI:** 10.1038/s41598-017-07097-9

**Published:** 2017-08-07

**Authors:** S. Lichtmannecker, M. Florian, T. Reichert, M. Blauth, M. Bichler, F. Jahnke, J. J. Finley, C. Gies, M. Kaniber

**Affiliations:** 1 Walter Schottky Institut and Physik Department, Technische Universität München, Am Coulombwall 4, 85748 Garching, Germany; 2 Institut für Theoretische Physik, Universität Bremen, Otto-Hahn-Allee 1, 28359 Bremen, Germany

## Abstract

We report on non-conventional lasing in a photonic-crystal nanocavity that operates with only four solid-state quantum-dot emitters. In a comparison between microscopic theory and experiment, we demonstrate that irrespective of emitter detuning, lasing with ${g}^{\mathrm{(2)}}=1$ is facilitated by means of emission from dense-lying multi-exciton states. In the spontaneous-emission regime we find signatures for radiative coupling between the quantum dots. The realization of different multi-exciton states at different excitation powers and the presence of electronic inter-emitter correlations are reflected in a pump-rate dependence of the *β*-factor.

## Introduction

Self-assembled semiconductor quantum dots (QDs) exhibit a discrete density of electronic states due to quantization effects owed to their nanometer-sized dimension^1^. In analogy to real atoms, QDs are often termed *artificial atoms* and are well-established in numerous research fields, such as solid-state cavity quantum electrodynamics (QED), photonic quantum technologies, and semiconductor lasers. The incorporation of QDs into high quality factor (Q) and low mode volume (V) optical microcavities^2^, like photonic crystals (PhCs)^3^, enables the exploration of the miniaturization limit of lasing, where the optical gain medium consists of a few, or even a single quantum emitter coupled to a single cavity mode^4–7^. In this regime, the unique electronic structure of each solid-state emitter matters and differences to natural few-atom lasers become apparent. Highly excited individual QDs possess a rich emission spectrum consisting of a multitude of close-lying (meV) discrete neutral and charged multi-exciton states^1^ that have been demonstrated to couple to the cavity mode even if they are not in perfect resonance^4, 8, 9^. Recently, another effect has been explored in atomic^10^ and solid-state lasers with ensembles of QDs^11^, in which few discrete emitters can exchange photons via a high-Q cavity mode, thereby establishing electronic inter-emitter correlations that are connected to superradiance. Analyzing the properties of few-QD nanocavity systems beyond the limitations of conventional laser models for independently acting atomic emitters can provide new insight into the fascinating working principles of cavity-QED driven nanolasers.

Here, we study a PhC nanocavity laser with an energy-tunable mode, pumped predominantly via the discrete multi-exciton states of only four QD emitters by combining confocal and photon correlation spectroscopy with a quantum-optical theory. The microscopic approach accounts for the semiconductor properties of the solid-state emitters by allowing multi-excitonic states of each emitter to couple to the cavity mode, and to form electronic correlations between different emitters. We identify non-resonant coupling as the underlying mechanism for lasing over a wide range of spectral detunings up to ${\rm{\Delta }} \sim 17\,$ meV between emitter and cavity mode. Evidence for lasing is based on measurements and calculations of the autocorrelation function ${g}^{\mathrm{(2)}}(\tau =\mathrm{0)}=(\langle {n}^{2}\rangle -\langle n\rangle )/{\langle n\rangle }^{2}$, with *n* being the number operator for photons in the laser mode. Furthermore, we find signatures of electronic correlations that are established between the emitters via the common light field of the PhC cavity mode. These correlations are not related to stimulated emission, which similarly establishes phase correlations in the presence of a sizable photon population in the mode^10^. Our theory accounts for these effects and predicts a strongly reduced spontaneous emission rate and super-thermal photon bunching with ${g}^{\mathrm{(2)}}(\tau =\mathrm{0)} > 2$. Due to pump-rate dependent realizations of different multi-exciton states that contribute to the emission and the presence of inter-emitter correlations, the system cannot be described by a single-valued *β*-factor, which is widely used as key characteristic of nanolaser devices^4, 6, 12^. Instead, *β* itself becomes pump-rate dependent. With the combination of experiment and microscopic theory, we are able to provide insight into few-emitter solid-state lasing that will aid the design of future highly efficient, low threshold nanolasers.

**Figure 1:**
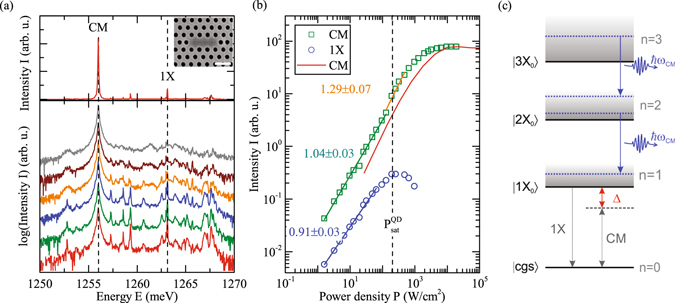
(**a**) Photoluminescence of the QD-cavity system (upper panel) and power-dependent measurement for power densities from 0.14 kW/cm^2^ to 5.9 kW/cm^2^ (lower panel). The spectra are plotted on a logarithmic scale with offset to each other for clarity. The cavity mode is labeled CM, a dominant QD-transition line 1X. Inset: Scanning electron microscope image of PhC nanocavity. Scale bar, 500 nm. (**b**) Integrated intensity of the cavity mode (green) and the QD 1X line (blue) as a function of excitation power density. Solid blue and green lines are power-law fits to the emission data. The solid red line depicts the intensity of the cavity mode emission calculated from theory. To connect the theoretical pump rate with the experimental power density, the red curve has been shifted along the power axis to ensure that the calculated exciton saturation coincides with ${P}_{{\rm{sat}}}^{{\rm{QD}}}$. (**c**) Schematic level scheme of the manifolds of multi-excitonic emission channels with $n=0\ldots 3$ excitations. The blue arrows indicate resonant recombination processes.

The investigated samples are free-standing GaAs membranes, loaded with a single layer of InGaAs QDs at its centre (areal density ~20 *μ*m^−2^) and embedded into two-dimensional PhCs with L3 line defect nanocavities^13^ (Further details on sample growth and fabrication are given in the Supplementary Information). The inset of Fig. 1(a) shows a representative scanning electron microscope image of this structure. A typical micro-photoluminescence (*μ*-PL) spectrum of a QD-cavity system subjected to resonant excitation via a higher-order cavity mode^14^ is shown in the upper panel of Fig. 1(a). The spectrum shows the fundamental (CM) cavity-mode emission at ${E}_{{\rm{CM}}}=1257.1$ meV with $Q=\mathrm{12,000}$ and several emission lines stemming from few (*N* = 4) QDs located in the PhC nanocavity, the strongest one at ${E}_{1X}=1263.1\,$ meV is labelled with 1*X*. Cavity-resonant excitation via a higher order mode guarantees that predominantly QDs inside the defect region are excited quasi-resonantly^14^.

In order to test the system for lasing, we first recorded *μ*-PL spectra as a function of excitation power density *P*, as shown on a semi-logarithmic scale in the lower panel of Fig. 1(a). The corresponding input-ouput curves of *CM* (squares) and 1*X* (circles) are shown on a double-logarithmic scale in Fig. 1(b). The dominant emission line, labelled 1*X*, stems from a single QD, confirmed by second-order photon correlation measurements ${g}_{1X}^{\mathrm{(2)}}(\tau =\mathrm{0)}$, where we obtain a reduced multi-photon emission probability of ${g}_{1X}^{\mathrm{(2)}}\mathrm{(0)}=0.25\pm 0.16$ ^6, 15^ (shown in the Supplementary Material). The weaker emission lines visible at lowest pump power are most likely either stemming from charged excitons of the same QD or from single exciton transitions of other QDs located inside the cavity region. The input-output curves are fitted by a power law $I=A\cdot {P}^{m}$, where *I* and *P* denote the intensity and pump power density, respectively. We observe an exponent ${m}^{{\rm{1X}}}=0.91\pm 0.03$ for the 1X-emission, indicating excitonic character^16^, before it saturates at ${P}_{{\rm{sat}}}^{{\rm{1X}}}=0.14\pm 0.1$  kW/cm^2^. The CM-emission is mainly determined via non-resonant cavity feeding^17, 18^ from the 1*X*-emission despite the large detuning of ${{\rm{\Delta }}}_{0} \sim -7$ meV and, thus, exhibits a similar slope ${m}_{1}^{{\rm{CM}}}=1.06\pm 0.02$ below ${P}_{{\rm{sat}}}^{{\rm{1X}}}$. This is further confirmed by second-order photon correlation measurements between 1*X* and *CM*, ${g}_{1X-CM}^{\mathrm{(2)}}(\tau )$ (i.e. cross-correlation) shown in the Supplementary Material. For ${P}_{{\rm{sat}}}^{{\rm{1X}}} < P < {P}_{{\rm{sat}}}^{{\rm{CM}}}$ we observe a slight superlinear increase of *CM* (${m}_{2}^{{\rm{CM}}}=1.29\pm 0.07$, highlighted in orange in Fig. 1(b)), which starts at ${P}_{{\rm{sat}}}^{{\rm{1X}}}$ of the QD when multi-exciton states become increasingly populated with significant probability. Most remarkably, we observe for $P > {P}_{{\rm{sat}}}^{{\rm{CM}}}=4.7\pm 0.4$ kW/cm^2^ a complete saturation of *CM*, strongly suggesting the suppression of non-saturable background contributions, such as 0D–2D transitions between QD and wetting layer states^19^. This is in contrast to QD-lasers with shallow confinement^4^ and reflects the finite gain provided by the multi-exciton states in the few-QD nanolaser presented here. The moderate kink in the input-ouput curve of *CM* combined with the saturation behaviour leads to a slightly s-shaped curve, which we attribute to ultra-low threshold lasing^4, 20^, a conclusion supported by the measurements presented below. We note that an unpronounced kink in the input-output characteristic as observed in our study makes it usually difficult to unambiguously claim lasing for the device. Therefore, we suggest that further advanced spectroscopy techniques, such as first and second order correlation measurements should be applied in order to test the device under study for coherent light emission.

To support our experimental findings and our assignment of lasing, we evaluate a theoretical model that accounts for the key physical elements, namely the multi-exciton states of the QD emitters, and their light-matter interaction with photons in the cavity mode. Strong excitation is typically required to drive a laser across the threshold. In this regime, multiple carriers accumulate in the QD so that a multitude of many-particle states arises due to the Coulomb configuration interaction. An illustration is given in Fig. 1(c). In terms of the number *n* of excitations (e-h pairs), for the *n* = 1 to *n* = 0 transition only well-separated emission lines exist, whereas for higher manifolds (gray shaded regions) dense-lying sets of transitions form, which are observed in the experiment as a broadband background (visible in Fig. 1(a)). Some of these transitions can be in resonance with the mode even though the exciton-to-ground-state transition is detuned by several meV^8, 9^. While single-photon sources use the exciton-to-ground state transition, these higher lying transitions are the ones that drive the emission into the laser mode. Which ones are realized depends on the excitation level and can vary as a function of the pump power.

For the numerical evaluation, we select transitions from different manifolds that are in resonance with the mode and account for their interplay by calculating the dynamics of the density matrix of the coupled four-emitter-photon system. We numerically solve the von Neumann-Lindblad equation $\frac{\partial }{\partial \,t\,}\rho =-i[{H}_{{\rm{JC}}},\rho ]+ {\mathcal L} \rho$ with the Jaynes-Cummings interaction Hamiltonian ${H}_{{\rm{JC}}}=g{\sum }_{\alpha ,i}[{b}^{\dagger }{D}_{\alpha ,i}^{l}+b{({D}_{\alpha ,i}^{l})}^{\dagger }]$. ${H}_{{\rm{JC}}}$ describes the non-perturbative light-matter interaction between all dipole-allowed transitions and many-particle configurations $|{i}_{\alpha }\rangle \mathrm{,\ }|{l}_{\alpha }\rangle$ of QD *α*, which are represented by ${D}_{\alpha ,i}^{l}$, and the quantised field of the PhC nanocavity (${b}^{\dagger }$ denotes the creation operator for photons in the laser mode). Excitation and relaxation processes are accounted for via Lindblad terms ${\mathcal L}$. Further details and information on the numerical implementation are given in the Supplementary Material. The density operator $\rho$ yields the output-intensity and ${g}^{\mathrm{(2)}}\mathrm{(0)}$. Moreover, it accounts for all correlations between electronic and photonic degrees of freedom, enabling the study of radiative coupling effects in our system. The red curve in Fig. 1(b) shows the calculated input-output curve of the few-QD nanolaser, which is in excellent agreement with the experiment. In particular, it reflects the varied photon-emission contributions from different multi-excitonic emission channels that are in resonance with the mode at different pump rates. As our laser model explicitly accounts for four QD emitters, both the slope and the saturation of the emission are well reproduced.

**Figure 2:**
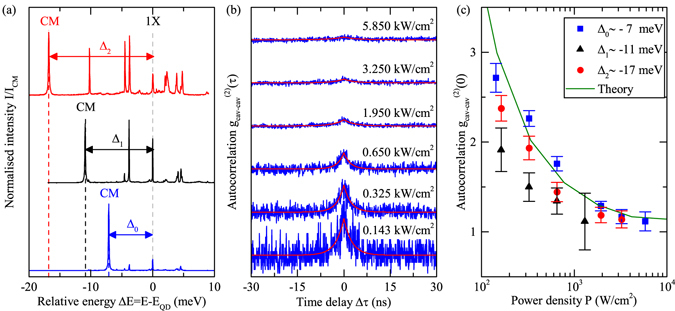
(**a**) Photoluminescence spectra for three different QD-CM detunings ${{\rm{\Delta }}}_{0} \sim -7$ meV (blue), ${{\rm{\Delta }}}_{1} \sim -11$ meV (black) and ${{\rm{\Delta }}}_{2}=-17$ meV (red). (**b**) Auto-correlation measurements of the cavity mode at ${E}_{{\rm{CM}}}$ for ${{\rm{\Delta }}}_{0}$ and excitation powers corresponding to spectra shown in Fig. 1(a) (lower panel). Solid lines are fits to the data. (**c**) Second oder correlation ${g}^{\mathrm{(2)}}\mathrm{(0)}$ as function of the excitation power density for ${{\rm{\Delta }}}_{0}$ (blue), ${{\rm{\Delta }}}_{1}$ (black) and ${{\rm{\Delta }}}_{2}$ (red). The solid green line has been obtained from the microscopic model.

To better understand the consequences of lasing via multi-exciton states, we vary the spectral cavity-mode position, and thereby the QD-cavity-mode detuning ${\rm{\Delta }}$, by local inert gas deposition onto the PhC^21, 22^. In Fig. 2(a), PL spectra are shown of the very same PhC nanocavity for detunings ${{\rm{\Delta }}}_{0}\simeq -7\,$ meV, ${{\rm{\Delta }}}_{1}\simeq -11$ meV and ${{\rm{\Delta }}}_{2}\simeq -17\,$ meV in blue, black and red, respectively. The photon autocorrelation function ${g}^{\mathrm{(2)}}\mathrm{(0)}$ has become the central tool for identifying the threshold in nanolasers, taking on the thermal (coherent) value of ${g}^{\mathrm{(2)}}\mathrm{(0)}=2$ (1) below (above) the threshold^12, 20, 23^. Unambiguous evidence for lasing at all detunings ${\rm{\Delta }}$ is provided by measuring the autocorrelation function of the $CM$-emission as a function of *P*. Zero-time-delay values are obtained from measured ${g}_{CM}^{\mathrm{(2)}}(\tau )$, shown in Fig. 2(b) for ${{\rm{\Delta }}}_{0}$, by fitting ${g}^{\mathrm{(2)}}(\tau )=1+A\cdot \exp (-2|\tau |/{t}_{0})$ (fits shown as solid lines)^15^. The resulting ${g}_{CM}^{\mathrm{(2)}}\mathrm{(0)}$ is shown in Fig. 2(c) (blue), together with the results for ${{\rm{\Delta }}}_{1}$ (black) and ${{\rm{\Delta }}}_{2}$ (red). For all ${\rm{\Delta }}$, with increasing *P* we observe a clear transition from ${g}_{CM}^{\mathrm{(2)}}\mathrm{(0)} > 1$ to coherent lasing with ${g}_{CM}^{\mathrm{(2)}}\mathrm{(0)}=1$, demonstrating a surprising robustness of our few-QD nanolaser with respect to spectral cavity-emitter detunings up to 17 meV due to efficient non-resonant coupling.

When compared to previous experiments, the pronounced bunching with ${g}_{CM}^{\mathrm{(2)}}\mathrm{(0)}$-values up to 2.7–exceeding the classical limit of 2 for thermal light^15^–strongly distinguishes the results in Fig. 2(c) from previous experimental and theoretical studies^4, 8, 20^. Recent theoretical work^11, 24, 25^ have identified ${g}^{\mathrm{(2)}} > 2$ as a fingerprint for a new regime of spontaneous emission with radiatively enhanced correlations between distant emitters mediated by the nanocavity. Our theoretical model accounts for the interplay of multi-excitonic emission channels between four QDs as well as their light-matter interaction with cavity photons and reproduces both, the super-thermal bunching, and the transition to lasing as shown by the solid line in Fig. 2(c) very well. The insight from microscopic theory allows us to attribute the enhanced two- and multiple-photon emission probability to two effects: (i) Competition between different resonant transitions in *each* QD enables simultaneous emission of photons into the mode, and (ii) strong radiative coupling between *different* emitters results in radiative coupling (subradiant regime). The latter effect has been reported recently under pulsed excitation in ref. 11 in accordance with earlier predictions^24, 26^.

**Figure 3:**
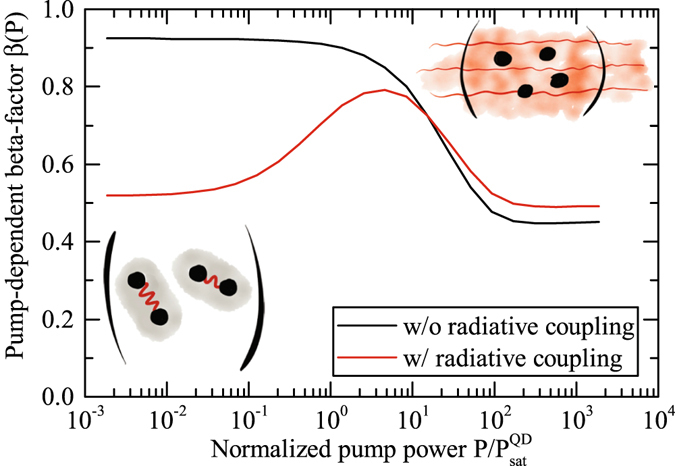
Pump-rate dependent *β*-factor obtained from the theoretical model with parameters applicable to the experimentally studied system (red curve). Calculation suppressing radiative coupling effects between emitters that are responsible for sub- and superradiant effects (black curve). Comparing both curves reveals that radiative coupling effects lead to a strong inhibition of spontaneous emission at low excitation (i.e. subradiance) and a slight enhancement of spontaneous emission above the laser threshold (i.e. superradiance).

Finally, we demonstrate that nanolasers containing only few, discrete quantum emitters as gain medium are not well characterised by the conventional spontaneous emission coupling factor *β*. Typically, *β* is obtained from rate equations for two-level systems^27^ that quantifies the fraction of the total spontaneous emission into the laser mode, $\beta ={\gamma }_{{\rm{l}}}/({\gamma }_{{\rm{l}}}+{\gamma }_{{\rm{nl}}})$, ${\gamma }_{{\rm{l}}}$ and ${\gamma }_{{\rm{nl}}}$ denoting the emission rates into lasing and non-lasing modes, respectively. According to the observation (i), in our few-QD nanolaser multi-exciton transitions from different emitters tune in and out of resonance with the cavity mode as pumping is varied, which is beyond the two-level approximation. Furthermore, the rate-equation approach assumes that all emitters act individually, which is in contrast to our observation (ii) of super-thermal emission as a *collective effect*. Therefore, we account for the varying coupling efficiency into the lasing mode for each of the multi-exciton emission channels by introducing a pump-rate dependent factor $\beta (P)=\frac{{\rm{\Gamma }}(P)}{{\rm{\Gamma }}(P)+{{\rm{\Gamma }}}_{{\rm{nl}}}(P)}$, where the spontaneous emission rate into the cavity mode ${\rm{\Gamma }}(P)$ and into non-lasing modes ${{\rm{\Gamma }}}_{{\rm{nl}}}(P)$ contain contributions from all bright multi-exciton configurations that are realized at a certain pump rate in an averaged form. To assess the impact of (i), we plot in Fig. 3 $\beta (P)$ without radiative coupling effects (black curve), where the asymptotic values at low ($\beta (P) > \mathrm{90 \% }$) and high ($\beta (P)\approx \mathrm{50 \% }$) excitation reflect the conventional *β*-factor associated with the transitions that dominate at low and high excitation. For intermediate pump powers, we observe a transition as the system switches between multi-excitonic emission channels. We note that often a kink in the input-output curve is used to quantify a constant *β*, which for few-QD nanolasers may actually result from transitions between multi-exciton states of various emitters tuning in and out of resonance at different excitation powers.

To assess the impact of (ii), we quantify the role of radiative coupling effects from their contribution to the spontaneous emission rate ${\rm{\Gamma }}(P)$ defined as^24^

1 $$\begin{array}{ccc}{\rm{\Gamma }}(P) & = & \sum _{i,j}[\sum _{\alpha =1}^{{N}_{{\rm{Q}}{\rm{D}}}}{R}_{i}(P)\langle {({D}_{\alpha ,i}^{l})}^{\dagger }{D}_{\alpha ,j}^{l}\rangle {\delta }_{i,j}+\sum _{\alpha \ne \beta }^{{N}_{{\rm{Q}}{\rm{D}}}}{R}_{i}(P)\langle {D}_{\alpha ,i}^{l}{({D}_{\beta ,j}^{l})}^{\dagger }\rangle ].\end{array}$$

Here, the operator ${D}_{\alpha ,i}^{l}$ describes an allowed (bright) dipole transition between multi-exciton states in QD *α*, with the initial state $|{i}_{\alpha }\rangle$ and the corresponding recombination rate ${R}_{i}(P)$. The quantum-mechanical average is taken with respect to the steady-state density operator. The expression in the brackets contains two contributions: The first summation accounts for the spontaneous-emission contributions from all QDs *α individually*. The second sum is the contribution of dipole-correlated transitions *in different emitters α* and *β* that arises due to radiative coupling. While this second contribution has been neglected for the black curve in Fig. 3, the red curve shows $\beta (P)$ including radiative coupling. In the low-excitation regime, where the super-thermal bunching is observed in ${g}_{CM}^{\mathrm{(2)}}\mathrm{(0)}$, inter-emitter coupling leads to a strong inhibition of the spontaneous emission rate and of the $\beta (P)$-factor by nearly a factor of 2. This reduced photon output is caused by the build-up of dipole-correlations between different emitters, which has been discussed in the context of superradiant lasing in an atomic system^10^ and for QD-micropillar lasers under pulsed excitation^11^. At $P\mathop{ > }\limits_{ \tilde {}}20{P}_{sat}^{QD}$ the spontaneous emission becomes enhanced as the second term in Eq. (1) changes sign and the radiative coupling changes from *sub-* to *super*-radiance^24, 26^. The enhancement in the high-excitation regime is smaller in comparison to the suppression at low excitation due to stimulated emission, which overrules the effect. Further details on the theoretical description can be found in the Supplementary Material.

In summary, we present new insight into the extraordinary operational regime of a few (~4) QD PhC nanolaser that operates solely by non-resonant coupling of QD multi-exciton transitions to the cavity mode. In a systematic study we demonstrate that this mechanism gives the laser emission a surprising stability against spectral detuning of the mode up to 17 meV. On the basis of a microscopic theory and ${g}^{\mathrm{(2)}}\mathrm{(0)}$ measurements exhibiting super-thermal values of up to 2.7, we find strong evidence that the spontaneous-emission regime is subject to radiative coupling between emitters in the form of subradiance. In the presence of such effects, few-emitter cavity-QED lasers are not well characterized by the conventional single-valued *β*-factor. A factor $\beta (P)$ is suggested that is pump-rate dependent and strongly determined by radiative coupling especially in the low-excitation regime, shining new light on the practice to determine *β* from the shape of the input-output curve alone.

## Electronic supplementary material

Suppl info
