# Supplementary material for: A few-emitter solid-state multi-exciton laser
Source: Sci Rep. 2017 Aug 7;7:7420. doi: 10.1038/s41598-017-07097-9 (PMC5547160; doi:10.1038/s41598-017-07097-9)
Supplement: Supplementary file 1 — Suppl info [file 41598_2017_7097_MOESM1_ESM.pdf]

Supplementary Material: A few-emitter solid-state multi-exciton laser

S. Lichtmannecker,[1] M. Florian,[2] T. Reichert,[1] M. Blauth,[1]
M. Bichler,[1] F. Jahnke,[2] J. J. Finley,[1] C. Gies,[2,*] and M. Kaniber[1,†]
[1] *Walter Schottky Institut and Physik Department, Technische Universität München, Am Coulombwall 4, 85748 Garching, Germany*
[2] *Institut für theoretische Physik, Universität Bremen, Otto-Hahn-Allee 1, 28359 Bremen*
(Dated: June 19, 2017)

FABRICATION AND EXERIMENTAL DETAILS

The sample investigated was grown using molecular beam epitaxy on a 350 µm thick [100] GaAs wafer. A 300 nm GaAs buffer layer was grown, followed by an 800 nm thick sacrificial layer of $Al_{0.8}Ga_{0.2}As$ and an 150 nm thick nominally undoped GaAs slab that contained a single layer of nominally $In_{0.5}Ga_{0.5}As$ QDs at its midpoint. The growth conditions used for the QD layer yielded an areal density $\rho_D \sim 20\ \mu\text{m}^{-2}$, emitting over the spectral range of $1270 - 1400$ meV. The average number of QDs has been evaluated via atomic force microscopy measurements on another sample, where the QDs have been grown under nominal identical growth conditions, however, without the GaAs capping layer. After growth, a hexagonal lattice of air holes was defined by electron beam lithography with a lattice constant of $a = 270$ nm in a ZEP 520-A soft mask and deeply etched using a $SiCl_4$ based inductively coupled plasma to form a 2D PhC [1]. We incorporated an optimized L3 cavity design [2, 3], giving rise to cavities with $V_{mode} \sim 0.92(\lambda/n)^3$ and $Q = 8000 - 15000$. In a final process step the AlGaAs layer was selectively removed with hydrofluoric acid to establish a free standing membrane. Taking into account the mode volume of the optimised L3 nanocavity design and the average areal QD density, one obtains on average ~ 3 QDs inside the cavity. Considering the broad emission bandwidth of 130 meV, the assumption of $N = 4$ QDs in the main manuscript represents a conservative upper estimation.

For optical studies the sample was mounted in a liquid He flow-cryostat and cooled to lattice temperatures of $T = 12$ K. Excitation of the sample was achieved via a 100× high numerical aperture $NA = 0.5$ confocal microscope objective that enables to focus light to a diffraction limit spot with $1/e^2$-size of 960 nm. The optical response from the system was collected via the same microscope objective and directly guided to a single imaging monochromator and detected with a liquid nitrogen cooled CCD camera. For measurements of the second order photon correlation function $g^{(2)}(\tau)$, the monochromator was used as a tunable bandpass filter with a bandwidth of 270 µeV. The spectrally filtered signal was coupled into a fiber-beamsplitter and guided to two separate avalanche photo diodes which provide single photon sensitivity and act as a Hanbury Brown and Twiss setup [4].

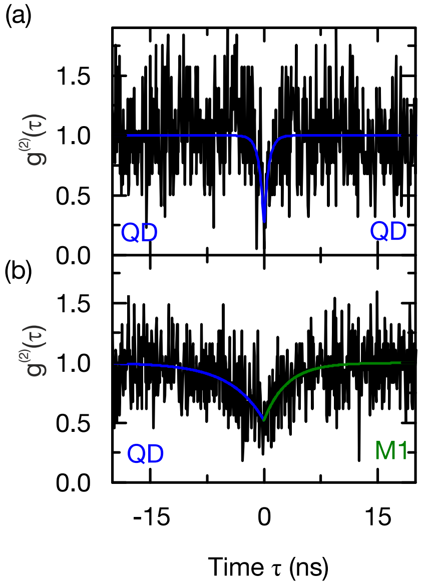

Figure SM 1. (a) Second-order autocorrelation measurement of QD emitting at $E_{\text{QD}} = 1263.1$ meV as shown in Figure 1a (main manuscript). (b) Second-order cross-correlation measurement between QD emitting at $E_{\text{QD}} = 1263.1$ meV and the cavitiy mode M1 emitting at $E_{\text{cav}} = 1257.1$ meV as shown in Figure 1a (main manuscript).

The avalanche photo diodes exhibit a temporal resolution of 350 ps. The detection events were time correlated using a Pico Quant TimeHarp time tagging module. For mode resonant excitation [5, 6] we used a continuous wave single frequency laser with a bandwidth of 100 kHz and a tuning range between 1259 meV and 1369 meV.

AUTO- AND CROSS-CORRELATION SPECTROSCOPY

To gain more insight into the coupling between the spectrally detuned QD and the cavity mode we performed measurements of the photon statistics of the emitted light. First we measured the second order photon correlation function $g^{(2)}(\tau)$ of the QD emitting at 1263.1 meV with an excitation power density close to saturation (~ 0.14 kW/cm^2) as shown in figure SM1 (a). For zero time delay $\tau = 0$ ns we observe a reduced number of correlation counts, giving rise to a value of

$g^{(2)}_{X-X}(0) = 0.25 \pm 0.16$, demonstrating the non-classical character of the studied quantum light and indicating that the signal stems pre-dominantly from a single quantum emitter [7, 8]. As we have proven the single photon characteristics of the emission we investigated the coupling of the QD to the fundamental cavity mode M1 by performing cross- correlation measurements between them. The measurement of the cross-correlation function $g^{(2)}_{X-cav}(\tau)$ is presented in figure SM1 (b) for the same excitation power density of $\sim 0.14\,\mathrm{kW/cm^2}$. As for the autocorrelation measurement of the QD, the measurement between QD and cavity mode yields a strong suppression of correlations for zero time delays with a $g^{(2)}_{X-cav}(0) = 0.52 \pm 0.07$. This antibunching unambiguously proves that the investigated QD is efficiently coupled to the cavity mode, since we would expect an uncorrelated constant statistic $g^{(2)}_{X-cav}(\tau) = 1$ for an uncoupled QD and cavity [9–11]. This non-resonant cavity mode feeding has been shown to be due to a number of mechanisms including coupling to acoustic phonons [12] and higher excited QD transitions [13]. The slightly increased value of $g^{(2)}_{X-cav}(0) = 0.52 \pm 0.07$ is attributed to additional sources of cavity feeding, most probably due to other spectrally detuned QD transitions. The different lifetimes $\tau^{QD}_{auto} = 0.61 \pm 0.19$ ns and $\tau^{QD}_{cross} = 3.0 \pm 0.65$ ns, extracted from the auto-correlation and cross-correlation measurement in figure SM1 (a) and (b), respectively, are due to different spectral cavity mode-QD detunings $\Delta^{cav-QD}_{auto} = 6.1$ meV and $\Delta^{cav-QD}_{cros} = 7.7$ meV, respectively. We conclude that we are investigating a state of the art nano-cavity doped with few single photon emitters that are efficiently non-resonantly coupled to the cavity mode via their multi-exciton states. Complete saturations of the cavity mode emission proves that the cavity mode is only pumped by a few QD states.

THEORETICAL METHODS

In this section we discuss the theoretical model underlying the results shown in figure 2 (main manuscript) and provide details about the calculation of the $\beta(P)$-factor presented in figure 3 (main manuscript). For the theoretical description we consider an ensemble of $N_{\mathrm{QD}} = 4$ self-assembled QDs coupled to a single high-quality mode of the PhC cavity. Each QD α possesses a multitude of many-particle states $|i_\alpha\rangle$, represented by different excitation manifolds n, where n stands for the total excitation number of e-h-pairs in the QD (compare figure 1 in the main manuscript). From these we choose six states ($|1\rangle \ldots |6\rangle$) that are numbered in order of increasing energy, as depicted in figure SM2. Optical transitions take place between states of manifolds n and $n-1$ that differ by one e-h-pair, and we assume that two transitions ($|6\rangle \to |5\rangle$ and $|2\rangle \to |1\rangle$) are optically bright and resonant with the laser mode. A special role takes on the transition from the $n = 1$ manifold to the ground state. In the experiment to which the model is applied, the ground-state exciton is strongly detuned from the cavity mode. In the configuration dynamics, the realization of the ground state is therefore strongly inhibited due to pumping, and the light-matter interaction is determined by the interplay of many-particle transitions between higher manifolds with $n > 0$. Therefore, the $n = 0$ state is not included in the calculations.

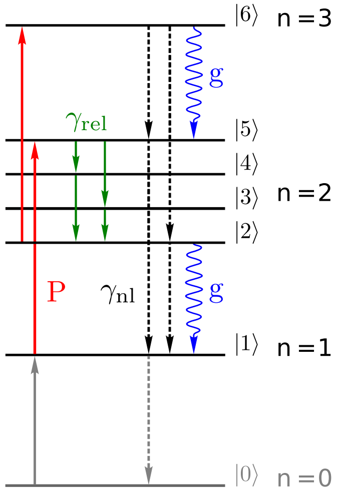

Figure SM 2. Schematic representation of the electronic QD level structure, corresponding to figure 1 (main manuscript) Light-matter coupling leads to recombination processes between the excitation manifolds with the light-matter coupling strength g. The arrows indicate the excitation, relaxation processses, and spontaneous emission into leaky modes taking place at a rate P, γ_{rel} and γ_{nl}, respectively. Transitions between $n = 1$ and $n = 0$ are strongly off-resonant, thus the dynamics of the $|0\rangle$ state is effectively described by $|1\rangle$, see text.

The Hilbert space of the multi-QD and cavity-photon system is spanned by the product states $|i_1\rangle \cdots |i_{N_{\mathrm{QD}}}\rangle\,|N\rangle$, where $|N\rangle$ defines the N-photon Fock state of the cavity mode. Considering the QDs and the photon mode as an open quantum system, one can describe the dynamics of the system density operator by the von Neumann-Lindblad (vNL) equation

$$\frac{\partial}{\partial t}\rho = -i[H_{\mathrm{JC}}, \rho] + \mathcal{L}\rho. \tag{1}$$

The coherent dynamics is represented by the commutator with the Jaynes-Cummings (JC) Hamiltonian

$$H_{\mathrm{JC}} = g \sum_{\alpha,i} \left[b^\dagger D^l_{\alpha,i} + b\left(D^l_{\alpha,i}\right)^\dagger \right], \tag{2}$$

describing the non-perturbative light-matter interaction between the QD-interband transitions and the quantized

field of the microcavity mode. Here, the operator

$$D^l_{\alpha,i} = \sum_j |\alpha, j\rangle \langle \alpha, i| \quad (3)$$

expresses all dipole-allowed transitions of QD α between many-particle state $|i_\alpha\rangle$ and $|j_\alpha\rangle$ that are resonant with the laser mode, i.e. $|6\rangle \to |5\rangle$ and $|2\rangle \to |1\rangle$. $b^\dagger$ and b are the bosonic creation and annihilation operators for photons in the laser mode, and g is the light-matter coupling strength for the respective electronic states and the cavity mode.

QDs are embedded systems, and their dynamics is strongly influenced by the coupling between localized QD states and the (quasi-) continuum of delocalized states and photon modes. While the former facilitates efficient carrier scattering into and within the QD via carrier–Coulomb and carrier–phonon interaction, the coupling to the latter leads to carrier recombination due to spontaneous emission. The incoherent, dissipative evolution results from the system-bath interaction, which leads to the sum of Lindblad operators

$$\mathcal{L}\rho = \sum_X \frac{\gamma_X}{2}\left[2X\rho X^\dagger - X^\dagger X\rho - \rho X^\dagger X\right]. \quad (4)$$

The operator X describes a reservoir-assisted transition in the system. The relevant information about the bath and its interaction with the system is contained in the transition rate γ_X. In the system we consider, X can be either a transition operator $|i_\alpha\rangle \langle j_\alpha|$ between two QD many-particle states, or a photonic annihilation operator b. The first case corresponds to a change from many-particle state $|j_\alpha\rangle$ to $|i_\alpha\rangle$, including pump excitation, spontaneous recombination into non-lasing modes and relaxation processes at rates P, γ_{nl} and γ_{rel}, respectively. All occurring incoherent processes are schematically depicted in figure SM2. In the second case the photon subsystem undergoes a lowering of the photon number due to cavity losses at a rate κ.

The von Neumann-Lindblad equation (1) is solved numerically until the steady-state solution is reached. Then various steady-state expectation values, such as the level populations and the photon statistics can be obtained. For the calculations shown in the main text, a light matter coupling strength of $g = 0.11/\mathrm{ps}$ is used, corresponding to a vacuum rabi splitting of $140\,\mu$eV. For the intraband relaxation rates γ_{rel} we use 0.5/ps. Radiative losses into non-lasing modes are strongly suppressed in photonic crystal cavity devices, and we consider $\gamma_{\mathrm{nl}} = 0.01/\mathrm{ps}$. The cavity decay rate of $\kappa = 0.16/\mathrm{ps}$ corresponds to a cavity-Q of 12000 in the spectral range of the InGaAs QD emission.

To calculate the pump-rate dependent spontaneous-emission coupling factor

$$\beta(P) = \frac{\Gamma(P)}{\Gamma(P) + \Gamma_{\mathrm{nl}}(P)} \quad (5)$$

that is described by the ratio of the spontaneous emission into the lasing mode and the total spontaneous emission, at a given pump rate P we consider the equation of motion for the cavity mean photon number

$$\frac{d}{dt}\langle b^\dagger b\rangle = -\kappa \langle b^\dagger b\rangle + \Gamma + \Gamma_{\mathrm{stim}}. \quad (6)$$

It is determined by the balance between cavity losses in the first term and photon emission. The second term constitutes the total spontaneous emission into the laser mode

$$\Gamma = \Gamma_{\mathrm{spont}} + \Gamma_{\mathrm{sr}}. \quad (7)$$

Here Γ_{spont} is the usual contribution from independent emitters to the spontaneous emission, and Γ_{sr} reflects the enhancement or suppression of spontaneous emission due to QD-QD correlations. The third term in Eq. (6) represents the contribution due to stimulated emission and absorption.

In the steady state we obtain

$$\Gamma_{\mathrm{spont}} = \sum_{\alpha=1}^{N_{\mathrm{QD}}} \sum_i R_i \left\langle \left(D^l_{\alpha,i}\right)^\dagger D^l_{\alpha,i} \right\rangle \quad (8)$$

and

$$\Gamma_{\mathrm{sr}} = \sum_{\alpha\neq\beta}^{N_{\mathrm{QD}}} \sum_{i,j} R_i \left\langle D^l_{\alpha,i} \left(D^l_{\beta,j}\right)^\dagger \right\rangle. \quad (9)$$

For more details we refer to Ref. [14]. The quantity R_i is the spontaneous emission rate for recombination of the many-particle state $|i_\alpha\rangle$

$$R_i = \frac{4g^2}{\kappa + \gamma_i^{\mathrm{tot}}}, \quad (10)$$

which resembles the form known from rate equations, where γ_i^{tot} is the total dephasing of the many-particle transition with the initial state $|i_\alpha\rangle$. Similarly, an expression for the emission into non-lasing modes can be found

$$\Gamma_{\mathrm{nl}} = \gamma_{\mathrm{nl}} \sum_{\alpha=1}^{N_{\mathrm{QD}}} \sum_i \left\langle \left(D^{\mathrm{nl}}_{\alpha,i}\right)^\dagger D^{\mathrm{nl}}_{\alpha,i} \right\rangle. \quad (11)$$

In this expression $D^{\mathrm{nl}}_{\alpha,i}$ describes all dipole-allowed transitions of QD α that are detuned from the laser mode. Eqns. (5) - (11) are explicitly evaluated to obtain the pump-power dependent $\beta(P)$-factor introduced in the main text. All averages are taken with respect to the steady state density matrix.

* gies@itp.uni-bremen.de

† michael.kaniber@wsi.tum.de

[1] A. Kress, F. Hofbauer, N. Reinelt, M. Kaniber, M. Bichler, D. Schuh, G. Boehm, and J. J. Finley, Proc. of SPIE **5733**, 114 (2005).
[2] T. Yoshie, A. Scherer, J. Hendrickson, G. Khitrova, H. M. Gibbs, G. Rupper, C. Ell, O. B. Shchekin, and D. G. Deppe, Nature **432**, 200 (2004).
[3] T. Asano and B. Song, Selected Topics in ... **12**, 1123 (2006).
[4] R. Hanbury Brown and R. Q. Twiss, Nature **177**, 27 (1956).
[5] M. Nomura, S. Iwamoto, T. Nakaoka, S. Ishida, and Y. Arakawa, Applied Physics Letters **88**, 141108 (2006).
[6] M. Kaniber, A. Neumann, A. Laucht, M. F. Huck, M. Bichler, M. C. Amann, and J. J. Finley, New Journal of Physics **11**, 013031 (2009).
[7] P. Michler, A. Kiraz, C. Becher, W. V. Schoenfeld, P. M. Petroff, L. Zhang, E. Hu, and Imamoglu, Science **290**, 2282 (2000).
[8] C. Santori, M. Pelton, G. Solomon, Y. Dale, and Y. Yamamoto, Physical Review Letters **86**, 1502 (2001).
[9] K. Hennessy, A. Badolato, M. Winger, D. Gerace, M. Atature, S. Gulde, S. Falt, E. L. Hu, and A. Imamoglu, Nature **445**, 896 (2007).
[10] D. Press, S. Götzinger, S. Reitzenstein, C. Hofmann, A. Löffler, M. Kamp, A. Forchel, and Y. Yamamoto, Physical Review Letters **98**, 117402 (2007).
[11] M. Kaniber, A. Laucht, A. Neumann, J. M. Villas-Bôas, M. Bichler, M. C. Amann, and J. J. Finley, Physical Review B **77**, 161303(R) (2008).
[12] U. Hohenester, A. Laucht, M. Kaniber, N. Hauke, A. Neumann, A. Mohtashami, M. Seliger, M. Bichler, and J. J. Finley, Physical Review B **80**, 201311(R) (2009).
[13] A. Laucht, M. Kaniber, A. Mohtashami, N. Hauke, M. Bichler, and J. J. Finley, Physical Review B **81**, 241302(R) (2010).
[14] H. A. M. Leymann, A. Foerster, F. Jahnke, J. Wiersig, and C. Gies, Physical Review Applied **4** (2015).
